# Covariate association eliminating weights: a unified weighting framework for causal effect estimation

**DOI:** 10.1093/biomet/asy015

**Published:** 2018-09-03

**Authors:** Sean Yiu, Li Su

**Affiliations:** Medical Research Council Biostatistics Unit, School of Clinical Medicine, University of Cambridge, Robinson Way, Cambridge CB2 0SR, U.K.

**Keywords:** Causal inference, Confounding, Continuous treatment, Covariate balance, Inverse probability weighting, Propensity function

## Abstract

Weighting methods offer an approach to estimating causal treatment effects in observational studies. However, if weights are estimated by maximum likelihood, misspecification of the treatment assignment model can lead to weighted estimators with substantial bias and variance. In this paper, we propose a unified framework for constructing weights such that a set of measured pretreatment covariates is unassociated with treatment assignment after weighting. We derive conditions for weight estimation by eliminating the associations between these covariates and treatment assignment characterized in a chosen treatment assignment model after weighting. The moment conditions in covariate balancing weight methods for binary, categorical and continuous treatments in cross-sectional settings are special cases of the conditions in our framework, which extends to longitudinal settings. Simulation shows that our method gives treatment effect estimates with smaller biases and variances than the maximum likelihood approach under treatment assignment model misspecification. We illustrate our method with an application to systemic lupus erythematosus data.

## 1 Introduction

Weighting methods are widely used to estimate causal treatment effects. The propensity function, the conditional probability of receiving treatment given a set of measured pretreatment covariates (Imai & van Dyk, 2004), features prominently in weighting methods. A natural choice of weights is a ratio of the marginal probability of treatment assignment and the propensity function, henceforth referred to as the stabilized inverse probability of treatment weights (Robins, 2000; Robins et al., 2000). Despite the appeal of weighting methods, problems arise when the propensity function is unknown. Hence weights are usually constructed using the stabilized inverse probability of treatment weight structure with an estimated propensity function, often obtained by maximum likelihood (Imbens, 2000; Robins et al., 2000), although other methods have been proposed (Lee et al., 2010). However, because these estimation procedures do not directly aim at the goal of weighting, which is to eliminate the association between a set of measured pretreatment covariates satisfying the conditions in § 2·1 and treatment assignment after weighting, a slightly misspecified propensity function model can result in badly biased treatment effect estimates (Kang & Schafer, 2007). Recently, this problem has motivated new weighting methods that optimize covariate balance, the covariate balancing weights (Graham et al., 2012; Hainmueller, 2012; McCaffrey et al., 2013; Imai & Ratkovic, 2014, 2015; Zhu et al., 2015; Zubizarreta, 2015; Chan et al., 2016; Fong et al., 2018). These weights can dramatically improve the performance of weighting methods, but there is a lack of a framework to generalize them to complex treatment types, such as semicontinuous or multivariate treatments, and even to longitudinal settings.

In this paper, we introduce covariate association eliminating weights, a unified framework for constructing weights with the goal being that a set of measured pretreatment covariates will be unassociated with treatment assignment after weighting. Our method can be used to estimate causal effects for semicontinuous, count, ordinal, or even multivariate treatments, and it extends to longitudinal settings. An example of estimating the direct effect of a time-varying treatment on a longitudinal outcome is provided in § 8. Utilizing the generality of the propensity function and its capacity to characterize covariate associations with treatment assignment, we derive conditions for weight estimation by eliminating the association between the set of measured pretreatment covariates and treatment assignment specified in a chosen propensity function model after weighting, i.e., by solving the weighted score equations of the propensity function model at parameter values which indicate that the covariates are unassociated with treatment assignment.

Our method has several attractive characteristics. First, it encompasses existing covariate balancing weight methods and provides a unified framework for weighting with treatments of any distribution; see § 4. By eliminating the associations between the covariates and treatment assignment after weighting, our method can provide some robustness against misspecification of the functional forms of the covariates in a propensity function model, particularly if they are predictive of the outcome; see § 6. Second, it is clear from our framework what type of covariate associations are eliminated after weighting. For example, the covariate balancing weight method proposed in Fong et al. (2018) will only eliminate the associations between the covariates and the mean of a continuous treatment; see § 4·2. Our method can also eliminate the associations between the covariates and the variance of the continuous treatment. Third, our method extends to longitudinal settings; see § 8. In particular, apart from handling treatments of any distribution, it can accommodate unbalanced observation schemes and can incorporate a variety of stabilized weight structures. In contrast, to the best of our knowledge, the only available covariate balancing weight method for longitudinal settings, proposed by Imai & Ratkovic (2015), focuses on binary treatments in a balanced observation scheme, and it is not clear how to incorporate arbitrary stabilized weight structures in their approach. Finally, our method can be implemented with standard statistical software by solving a convex optimization problem that identifies minimum-variance weights subject to our conditions (Zubizarreta, 2015). This is especially appealing for nonbinary treatments with outliers (Naimi et al., 2014), because it protects against extreme weights which often lead to unstable treatment effect estimates in practice.

## 2 The propensity function

### 2·1 Definition and assumptions

Let *X_i_*, *T_i_* and *Y_i_* be respectively a set of measured pretreatment covariates, the possibly multivariate treatment variable and the outcome for the *i*th unit (*i* = 1, … , *n*) in a simple random sample of size *n*. Following Imai & van Dyk (2004), we define the propensity function as the conditional probability of treatment given the set of measured pretreatment covariates, i.e., pr(*T_i_* | *X_i_*; *β*_true_), where *β*_true_ parameterizes this distribution. The parameter *β*_true_ is assumed to be unique and finite-dimensional, and is such that pr(*T_i_* | *X_i_*) depends on *X_i_* only through a subset of *β*_true_; this is the uniquely parameterized propensity function assumption of Imai & van Dyk (2004). For example, if pr(*T_i_* | *X_i_*) follows a regression model, then *β*_true_ would include regression coefficients that characterize the dependence of *T_i_* on *X_i_* and intercept terms that describe the baseline distribution of *T_i_*. In addition, we make the strong ignorability of treatment assignment assumption (Rosenbaum & Rubin, 1983), also known as the unconfoundedness assumption, pr{*T_i_* | *Y_i_*(*t*^P^), *X_i_*} = pr(*T_i_* | *X_i_*) where *Y_i_*(*t*^P^) is a random variable that maps a potential treatment *t*^P^ to a potential outcome, and the positivity assumption (Imai & van Dyk, 2004), $\text{pr}(T_{i}\in 𝒜|X_{i})>0$ for all *X_i_* and any set 𝒜 with positive measure. Finally, the distribution of potential outcomes for one unit is assumed to be independent of the potential treatment value of another unit given the set of pretreatment covariates; this is the stable unit treatment value assumption. Throughout the paper, we make the above assumptions; otherwise our method may result in severely biased causal effect estimates, even compared with an unadjusted analysis. For example, when unconfoundedness holds without conditioning on *X_i_*, adjusting for *X_i_* can induce M-bias (Ding & Miratrix, 2015).

### 2·2 Covariate selection

We briefly review some methods for covariate selection. When the causal structure is known and represented by a directed acyclic graph, Shpitser et al. (2010) gave a complete graphical criterion, the adjustment criterion, to determine whether adjusting for a set of covariates ensures unconfoundedness. The adjustment criterion generalizes the back-door criterion of Pearl (1995), which is sufficient but not necessary for unconfoundedness. In the absence of knowledge about how covariates are causally related to each other, VanderWeele & Shpitser (2011) proposed the disjunctive cause criterion. This says that if any subset of pretreatment covariates suffices to ensure unconfoundedness, then the subset of pretreatment covariates that are causes of the treatment assignment and/or the outcome will also suffice.

Given that an adjustment set that ensures unconfoundedness has been identified, many researchers have proposed dimension reduction procedures to increase efficiency while maintaining unconfoundedness (de Luna et al., 2011; VanderWeele & Shpitser, 2011), or to minimize mean squared error (Vansteelandt et al., 2012). Broadly, these methods tend to remove from the adjustment set covariates that are unassociated with the outcome.

### 2·3 Stabilized inverse probability of treatment weighting

A popular approach to causal effect estimation is to weight each unit’s data by stabilized inverse probability of treatment weights *W_i_* = *W_i_*(*T_i_*, *X_i_*) = pr(*T_i_*)/pr(*T_i_* | *X_i_*) (Robins et al., 2000). The idea is that if the propensity function is known, the propensity function after weighting by *W_i_*, pr*(*T_i_* | *X_i_*), will be equivalent to pr(*T_i_*) and hence does not depend on *X_i_*, as shown in the Supplementary Material. Here * denotes the pseudo-population after weighting. Under the assumptions in § 2·1, weighting by *W_i_* also preserves the causal effect of *t*^P^ on *E*{*Y_i_*(*t*^P^)} in the original population (Robins, 2000; Zhang et al., 2016), and so the causal effect can be consistently estimated without adjusting for *X_i_* in the weighted data. For example, *E*{*Y_i_*(*t*^P^)} can be consistently estimated by modelling *E*(*Y_i_* | *T_i_*) in the weighted data (Robins, 2000).

### 2·4 Maximum likelihood estimation

Estimating the weights by maximum likelihood involves specifying parametric models pr(*T_i_*; *α*) and $\text{pr}\left(T_{i}|{\tilde{X}}_{i};\beta \right),$ where ${\tilde{X}}_{i}$ are functionals of elements in *X_i_*, and then estimating the unknown parameters *α* and *β* by solving the score equations

$$S(\alpha )=\sum_{i=1}^{n}\frac{\partial }{\partial \alpha }\text{log}\;\text{pr}(T_{i};\alpha )=0,\;S(\beta )=\sum_{i=1}^{n}\frac{\partial }{\partial \beta }\text{log}\;\text{pr}(T_{i}\;|\;{\tilde{X}}_{i};\beta )=0.$$

If $\text{pr}\left(T_{i}|{\tilde{X}}_{i};\beta \right)$ and pr(*T_i_*; *α*) are correctly specified, then the weights $W_{i}=W_{i}\left(T_{i},{\tilde{X}}_{i};\hat{\alpha },\hat{\beta }\right)=\text{pr}\left(T_{i};\hat{\alpha }\right)/\text{pr}\left(T_{i}|{\tilde{X}}_{i};\hat{\beta }\right),$ where $\hat{\alpha }$ and $\hat{\beta }$ are the maximum likelihood estimates of *α* and *β*, are equivalent to $\text{pr}(T_{i})/\text{pr}(T_{i}|{\tilde{X}}_{i})$ asymptotically. Thus weighting by *W_i_* will result in ${\text{pr}}^{\text{*}}\left(T_{i}|{\tilde{X}}_{i}\right)$ being asymptotically equivalent to pr(*T_i_*), i.e., *T_i_* does not depend on ${\tilde{X}}_{i}$ after weighting. Here ${\text{pr}}^{\text{*}}\left(T_{i}|{\tilde{X}}_{i}\right)$ depends on $\hat{\alpha }$ and $\hat{\beta }$ through the estimated weights. However, when $\text{pr}\left(T_{i}|{\tilde{X}}_{i};\beta \right)$ is misspecified, this estimation procedure not only will result in ${\text{pr}}^{\text{*}}\left(T_{i}|{\tilde{X}}_{i}\right)$ diverging from pr(*T_i_*) but also does not even guarantee that the association between ${\tilde{X}}_{i}$ and *T_i_* is reduced in the weighted data relative to the observed data. Researchers are therefore encouraged to check for the absence of this association in the weighted data before proceeding to causal effect estimation. For nonbinary treatments, correctly specifying the propensity function model will generally also entail correct specification of the distribution and the dependence structure on covariates for higher-order moments of the treatment variable. Therefore, model misspecification for nonbinary treatments is likely to be worse.

## 3 Methodology

### 3·1 General framework

Maximum likelihood estimation indirectly aims to achieve the asymptotic equivalence of ${\text{pr}}^{\text{*}}\left(T_{i}|{\tilde{X}}_{i}\right)$ and pr(*T_i_*) by fitting a model $\text{pr}\left(T_{i}|{\tilde{X}}_{i};\beta \right)$ for the propensity function. We instead propose to use weighting to directly eliminate the association between ${\tilde{X}}_{i}$ and *T_i_* characterized by a chosen propensity function $\text{pr}\left(T_{i}|{\tilde{X}}_{i};\beta \right)$ in the weighted data. When $\text{pr}\left(T_{i}|{\tilde{X}}_{i};\beta \right)$ is misspecified, our method, in contrast to maximum likelihood estimation, will eliminate the association between ${\tilde{X}}_{i}$ and *T_i_* as characterized by $\text{pr}\left(T_{i}|{\tilde{X}}_{i};\beta \right)$ after weighting. This is necessary for *T_i_* to be independent of ${\tilde{X}}_{i}$ after weighting. In the unlikely scenario that $\text{pr}\left(T_{i}|{\tilde{X}}_{i};\beta \right)$ is correctly specified, maximum likelihood estimation will asymptotically eliminate the association between ${\tilde{X}}_{i}$ and *T_i_* after weighting, while our method will eliminate their association in finite samples.

We now formalize our ideas. Given a set of known weights *W* = (*W*_1_, … , *W_n_*), we can fit a parametric propensity function model $\text{pr}\left\{T_{i}|{\tilde{X}}_{i};\beta \left(W\right)\right\}$ to the data weighted by *W* by solving the score equations

(1) $$\sum_{i=1}^{n}W_{i}\frac{\partial }{\partial \beta (W)}\text{log}\;\text{pr}\{T_{i}|{\tilde{X}}_{i};\beta (W)\}=0,$$

where *β*(*W*) is a vector of parameters. Here we write *β*(*W*) as a function of *W* because the resulting maximum likelihood estimates, $\hat{\beta }\left(W\right),$ will depend on *W*. We use the uniquely parameterized propensity function assumption in § 2·1 to partition *β*(*W*) into {*β*_b_(*W*), *β*_d_(*W*)}, where *β*_d_(*W*) are the unique parameters that characterize the dependence of *T_i_* on ${\tilde{X}}_{i},$ e.g., regression coefficients, and *β*_b_(*W*) are parameters that characterize the baseline distribution, e.g., the intercept terms. Here the subscripts ‘d’ and ‘b’ stand for dependence and baseline, respectively. Without loss of generality, we assume $\text{pr}\left[T_{i}|{\tilde{X}}_{i};\left\{\beta_{\text{b}}\left(W\right),\beta_{\text{d}}\left(W\right)=0\right\}\right]=\text{pr}\left\{T_{i};\beta_{\text{b}}\left(W\right)\right\}.$ The conditions for covariate association eliminating weights are then derived by inverting (1) such that the weights *W* satisfy the equations

(2) $$\sum_{i=1}^{n}W_{i}\frac{\partial }{\partial \beta (W)}\text{log}\;\text{pr}\{T_{i}\;|\;{\tilde{X}}_{i};\beta (W)\}{|}_{\left\{\beta_{\text{b}}(W)=\hat{\alpha },\;\beta_{\text{d}}(W)=0\right\}}=0,$$

where $\hat{\alpha }$ is obtained by fitting pr(*T_i_*; *α*) to the observed data. Our method therefore sets the goal of weighting as attaining $\hat{\beta }\left(W\right)=\left\{{\hat{\beta }}_{\text{b}}\left(W\right)=\hat{\alpha },{\hat{\beta }}_{\text{d}}\left(W\right)=0\right\};$ that is, after weighting by *W_i_*, (i) *T_i_* is unassociated with ${\tilde{X}}_{i}$ as described by $\text{pr}\left\{T_{i}|{\tilde{X}}_{i};\beta \left(W\right)\right\}$ and (ii) the marginal distribution of *T_i_* is preserved from the observed data, as described by pr(*T_i_*; *α*). Here (ii) is a statement concerning the projection function, the treatment assignment distribution in the weighted data. The choice of projection function, as long as it does not depend on ${\tilde{X}}_{i},$ does not affect the consistency of weighted estimators for causal treatment effects, although it does affect their efficiency (Peterson et al., 2010). In our method, the projection function may be altered by fixing *β*_b_(*W*) in (2) at other values instead of $\hat{\alpha }$; an example is provided in § 4·1.

Our method is linked to the use of regression to assess covariate balance, for example in matched data (Lu, 2005). Specifically, if applying regression to matched data indicates small associations between the covariates and treatment assignment, it would be reasonable to assume that the covariate distributions are approximately balanced across treatment levels. Inspired by this, we propose to invert this covariate balance measure for weight estimation such that it implies no imbalances in covariate distributions across treatment levels, i.e., ${\hat{\beta }}_{\text{d}}\left(W\right)=0,$ after weighting.

### 3·2 Convex optimization for weight estimation

We consider a convex optimization approach in the spirit of Zubizarreta (2015), intended to estimate minimum-variance weights subject to the conditions (2) being satisfied. We formulate the estimation problem as a quadratic programming task and use the lsei function in the R (R Development Core Team, 2018) package limSolve (Soetaert et al., 2009) to obtain the optimal weights. Specifically, we solve the following quadratic programming problem for *W*:

(3) $$\text{minimize}\;\sum_{i=1}^{n}{\left(W_{i}-1\right)}^{2}$$

(4) $$\text{subject}\;\text{to}\;\sum_{i=1}^{n}W_{i}\frac{\partial }{\partial \beta (W)}\text{log}\;\text{pr}\{T_{i}\;|\;{\tilde{X}}_{i};\beta (W)\}{|}_{\left\{\beta_{\text{b}}(W)=\hat{\alpha },\;\beta_{\text{d}}(W)=0\right\}}=0,$$

(5) $$\sum_{i=1}^{n}W_{i}=n,\;W_{i}⩾0\;(i=1,\ldots ,n).$$

The constraints (4) are the equations (2), and so should eliminate the associations between covariates and treatment assignment after weighting. They also preserve the marginal distribution of the treatment variable in the observed data. The first constraint in (5) ensures equality of the numbers of units in the weighted and observed data. Since this also ensures that the mean of *W* is unity, (3) minimizes the variance of *W*. Interestingly, since the weights in the observed data are ones, (3) can also be interpreted as identifying the least extrapolated data as characterized by the *L*_2_-norm. The second constraint in (5) requires that each element of *W* be nonnegative (Hainmueller, 2012; Zubizarreta, 2015). Allowing some elements of *W* to be zero can let the estimation problem be formulated as a convex quadratic programming problem. Then the estimation procedure could remove units which contribute greatly to the variability of the weights, while forcing the remaining units to allow unbiased estimation of causal treatment effects (Crump et al., 2009). Following Zubizarreta (2015), it is possible to relax the strict equality constraints (4) to inequalities; the R function lsei has this option. This allows for less extrapolation at the expense of possibly introducing bias. For simplicity, we consider only the case where the strict equality constraints (4) are enforced.

## 4 Relation to previous methods

### 4·1 Binary treatment

The moment conditions specified in existing covariate balancing weight methods (Imai & Ratkovic, 2014; Fong et al., 2018) are special cases of the conditions (2) after slight modifications. In this section we focus on binary treatments. The details for more general categorical treatments can be found in the Supplementary Material.

Unless stated otherwise in our derivations, we implicitly take *β*_d_(*W*) and *β*_b_(*W*) in (2) to be, respectively, the vector of regression coefficients of ${\tilde{X}}_{i}$ and the vector of the remaining parameters, including intercept terms, in the chosen propensity function model.

Let $X_{i}^{*}={\left(1,{\tilde{X}}_{i}^{\text{T}}\right)}^{\text{T}}.$ When *T_i_* is a binary treatment variable, i.e., *T_i_* = 1 if the *i*th unit received treatment and *T_i_* = 0 otherwise, the following covariate balancing conditions for the estimation of the propensity score have been proposed (Imai & Ratkovic, 2014):

(6) $$\frac{1}{n}\sum_{i=1}^{n}W_{i}T_{i}X_{i}^{*}=\frac{1}{n}\sum_{i=1}^{n}W_{i}(1-T_{i})X_{i}^{*}.$$

Because $X_{i}^{*}$ includes 1, these covariate balancing conditions constrain the number of units in the treated and control groups to be equal in the weighted data. The other conditions for ${\tilde{X}}_{i}$ constrain the weighted means of each element in ${\tilde{X}}_{i}$ in the treated and control groups to be equal.

These conditions can be derived using our framework. Suppose that we specify a logistic regression model for the propensity function/score, $\text{pr}\left\{T_{i}=1\;|\;{\tilde{X}}_{i};\beta \left(W\right)\right\}=1/\left[1+\text{exp}\left\{-\beta {\left(W\right)}^{\text{T}}X_{i}^{*}\right\}\right],$ in the weighted data induced by a set of known weights *W*. This model can be fitted to the weighted data by solving the score equations

$$\sum_{i=1}^{n}W_{i}X_{i}^{*}\left[T_{i}-\frac{1}{1+\text{exp}\{-\beta {\left(W\right)}^{\text{T}}X_{i}^{*}\}}\right]=0.$$

Conditions can be derived by fixing $1/\left[1+\text{exp}\left\{-\beta {\left(W\right)}^{\text{T}}X_{i}^{*}\right\}\right]={\hat{\pi }}_{0},$ i.e., letting ${\hat{\beta }}_{\text{d}}\left(W\right)=0,$ in these weighted score equations, where ${\hat{\pi }}_{0}$ is the proportion of units that received treatment in the observed data, and then inverting these equations so that we are solving for *W*:

(7) $$\sum_{i=1}^{n}W_{i}X_{i}^{*}(T_{i}-{\hat{\pi }}_{0})=0.$$

The correspondence between (6) and (7) can then be established by changing the projection function from ${\hat{\pi }}_{0}$ to 1/2.

### 4·2 Continuous treatment

When *T_i_* is continuous on the real line, Fong et al. (2018) proposed the following covariate balancing conditions for weight estimation,

(8) $$\frac{1}{n}\sum_{i=1}^{n}W_{i}X_{i}^{*\text{c}}T_{i}^{\text{c}}=0,$$

where the superscript c denotes the centred version of the variable and $X_{i}^{*}={\left(1,{\tilde{X}}_{i}^{\text{T}}\right)}^{\text{T}}.$ We now derive these covariate balancing conditions using the proposed framework. First, we assume a simple normal linear model for the propensity function, $T_{i}|{\tilde{X}}_{i};\beta (W)∼N\{\beta {\left(W\right)}^{\text{T}}X_{i}^{*},\sigma^{2}\}.$ The score equations for this model in the weighted data are

$$\sum_{i=1}^{n}W_{i}X_{i}^{*}\left\{\frac{T_{i}-\beta {\left(W\right)}^{\text{T}}X_{i}^{*}}{\sigma^{2}}\right\}=0,\;\sum_{i=1}^{n}W_{i}\left[-1+\frac{{\left\{T_{i}-\beta {\left(W\right)}^{\text{T}}X_{i}^{*}\right\}}^{2}}{\sigma^{2}}\right]=0.$$

By inverting these score equations, we find weights *W* that satisfy

(9) $$\sum_{i=1}^{n}W_{i}X_{i}^{*}\left(\frac{T_{i}-{\hat{\mu }}_{0}}{{\hat{\sigma }}_{0}^{2}}\right)=0,\;\sum_{i=1}^{n}W_{i}\left\{-1+\frac{{\left(T_{i}-{\hat{\mu }}_{0}\right)}^{2}}{{\hat{\sigma }}_{0}^{2}}\right\}=0,$$

where ${\hat{\mu }}_{0}$ and ${\hat{\sigma }}_{0}^{2}$ are the sample mean and variance of *T_i_*. The first set of conditions in (9) is equivalent to the conditions (8), except that the $X_{i}^{*}$ are not necessarily centred. The usefulness of our framework can also be exemplified by the insight it gives into how conditions can be specified for the variance of *T_i_*. Specifically, suppose that we specify an alternative propensity function model $T_{i}|{\tilde{X}}_{i};\beta_{\mu }(W),\beta_{\sigma }(W)∼N\left[\beta_{\mu }{\left(W\right)}^{\text{T}}X_{i}^{*},\text{exp}\{2\beta_{\sigma }{\left(W\right)}^{\text{T}}X_{i}^{*}\}\right];$ that is, we allow the variance of *T_i_*, $\sigma_{i}^{2},$ to depend on ${\tilde{X}}_{i}$ with $\sigma_{i}=\text{exp}\{\beta_{\sigma }{\left(W\right)}^{\text{T}}X_{i}^{*}\}.$ For this model, the conditions for weight estimation are derived by setting the regression coefficient elements in *β_μ_*(*W*) and *β_σ_*(*W*) to zero in the score equations. This corresponds to solving the equations

(10) $$\sum_{i=1}^{n}W_{i}X_{i}^{*}\left(\frac{T_{i}-{\hat{\mu }}_{0}}{{\hat{\sigma }}_{0}^{2}}\right)=0,\;\sum_{i=1}^{n}W_{i}X_{i}^{*}\left\{-1+\frac{{\left(T_{i}-{\hat{\mu }}_{0}\right)}^{2}}{{\hat{\sigma }}_{0}^{2}}\right\}=0.$$

Thus, the additional conditions in (10) are designed to remove the association between ${\tilde{X}}_{i}$ and the variance of *T_i_*. More details can be found in § 6.

## 5 Other treatment types

Having demonstrated that our framework encompasses previously proposed work, we now widen its applicability by considering semicontinuous treatments, motivated by our application in § 7. Details about count treatments can be found in the Supplementary Material.

Semicontinuous variables are characterized by a point mass at zero and a right-skewed continuous distribution with positive support (Olsen & Schafer, 2001). Semicontinuous treatments are common in clinical settings because only treated patients will be prescribed a continuous dose of treatment; otherwise their dose will be recorded as zero (Moodie & Stephens, 2010). A common approach to modelling semicontinuous data is by using a two-part model, such as that in Olsen & Schafer (2001):

(11) $$\text{pr}(T_{i}\;|\;{\tilde{X}}_{i};\pi_{i},\mu_{i},\sigma_{i})={\left(1-\pi_{i}\right)}^{I(T_{i}=0)}{\left[\frac{\pi_{i}}{\sigma_{i}}\phi \left\{\frac{g(T_{i})-\mu_{i}}{\sigma_{i}}\right\}\right]}^{I(T_{i}>0)},$$

where $\pi_{i}=1/\left[1+\text{exp}\{-\beta_{\pi }{\left(W\right)}^{\text{T}}X_{i}^{*}\}\right],\mu_{i}=\beta_{\mu }{\left(W\right)}^{\text{T}}X_{i}^{*}$ and $\sigma_{i}=\text{exp}\{\beta_{\sigma }{\left(W\right)}^{\text{T}}X_{i}^{*}\}$ with $X_{i}^{*}={\left(1,{\tilde{X}}_{i}^{\text{T}}\right)}^{\text{T}},\phi (\cdot )$ is the standard normal density function, and *I* (·) is an indicator function. Here *g*(·) is a monotonic function included to make the normal assumption for the positive values of *T_i_* more tenable. The likelihoods for the binary and continuous components of the two-part model are separable, so the results in § 4 imply that the conditions based on (11) are

(12) $$\sum_{i=1}^{n}W_{i}X_{i}^{*}\{I(T_{i}>0)-{\hat{\pi }}_{0}\}=0,$$

(13) $$\sum_{i:T_{i}>0}W_{i}X_{i}^{*}\left\{\frac{g(T_{i})-{\hat{\mu }}_{0}}{{\hat{\sigma }}_{0}^{2}}\right\}=0,\;\sum_{i:T_{i}>0}W_{i}X_{i}^{*}\left[-1+\frac{{\left\{g(T_{i})-{\hat{\mu }}_{0}\right\}}^{2}}{{\hat{\sigma }}_{0}^{2}}\right]=0,$$

where ${\hat{\pi }}_{0},\;{\hat{\mu }}_{0}\;\text{and}\;{\hat{\sigma }}_{0}$ are maximum likelihood estimates of *π_i_*, *μ_i_* and *σ_i_* obtained by fitting (11), but without covariates, to the observed *T_i_*. The conditions (12) are derived from the score equations for the binary component and are equivalent to (7). The conditions (13) are derived from the score equations of the continuous component and are similar to (10). In our framework, the weights *W* are estimated by solving (12) and (13) simultaneously, whereas maximum likelihood estimation obtains weights *W*_b*i*_ and *W*_c*i*_ separately from the binary and continuous components, respectively, and then uses their unit-specific product *W_i_* = *W*_b*i*_*W*_c*i*_ as the final weight.

## 6 Simulation study

We consider the set-up where there are three independent standard normal pretreatment covariates *X*_1*i*_, *X*_2*i*_ and *X*_3*i*_. The treatment *T_i_* is semicontinuous. We first simulate a binary indicator for *T_i_* > 0 with pr(*T_i_* > 0) = 1/[1 + exp{−(0·5 + *X*_1*i*_ + *X*_2*i*_ + *X*_3*i*_)}]. Then if *T_i_* > 0, *T_i_* is drawn from a normal distribution with mean 1 + 0·5*X*_1*i*_ + 0·2*X*_2*i*_ + 0·4*X*_3*i*_ and standard deviation exp(0·3 + 0·3*X*_1*i*_ + 0·1*X*_2*i*_ + 0·2*X*_3*i*_). The outcome *Y_i_* follows a negative binomial distribution

(14) $$\text{pr}(Y_{i}=y_{i})=\frac{\Gamma (y_{i}+1/\theta )}{\Gamma (1/\theta )y_{i}!}{\left(\frac{\theta \lambda_{i}}{1+\theta \lambda_{i}}\right)}^{y_{i}}{\left(\frac{1}{1+\theta \lambda_{i}}\right)}^{1/\theta }\;(y_{i}=0,1,\ldots ),$$

where *θ* = 1 and *λ_i_* = *E*(*Y_i_* | *T_i_*, *X*_1*i*_, *X*_2*i*_, *X*_3*i*_) = exp[−1 + 0·5*T_i_* + 2/{1 + exp(−3*X*_1*i*_)} + 0·2*X*_2*i*_ − 0·2 exp(*X*_3*i*_)]. Using this set-up, we generate four sets of 2500 simulated datasets with *n* = 500, 1000, 2500 and 4000.

For each of the four sets of simulations, we use the proposed method for semicontinuous treatments in § 5, referred to as Approach 1, and maximum likelihood estimation, referred to as Approach 2, with the propensity function model (11) to obtain the weights. For both methods we consider two different model structures and two sets of covariates for the propensity function model. The correct model structure A allows the mean and variance of *T_i_* conditional on *T_i_* > 0 to depend on covariates, whereas the incorrect model structure B restricts *σ_i_* to be a constant *σ*. The first set of covariates are the correct covariates, ${\tilde{X}}_{i}={\left(X_{1i},X_{2i},X_{3i}\right)}^{\text{T}},$ and the second set are transformed covariates of the form ${\tilde{X}}_{i}={\left(X_{1i}^{t},X_{2i}^{t},X_{3i}^{t}\right)}^{T},\;\text{Where}\;X_{1i}^{t}={\left(1+X_{1i}+X_{2i}\right)}^{2},$ $X_{2i}^{t}=X_{2i}/\{1+\text{exp}(X_{1i})\}\;\text{and}\;X_{3i}^{t}={\left(X_{3i}\right)}^{3}.$ The covariates $X_{1i}^{t}\;\text{and}\;X_{3i}^{t}$ were chosen to be highly predictive of the outcome. In total, we fit eight models to each simulated dataset to estimate the weights, which are scaled by their averages so that they sum to *n*. Then, using the estimated weights, we fit a weighted negative binomial model (14) to the outcome with the marginal mean *λ_i_* = exp(*γ*_0_ + *γ*_1_*T_i_*), where *γ*_0_, *γ*_1_ and *θ* are parameters to be estimated. The true causal treatment effect of interest is *γ*_1_ = 0·5.

**Fig. 1:**
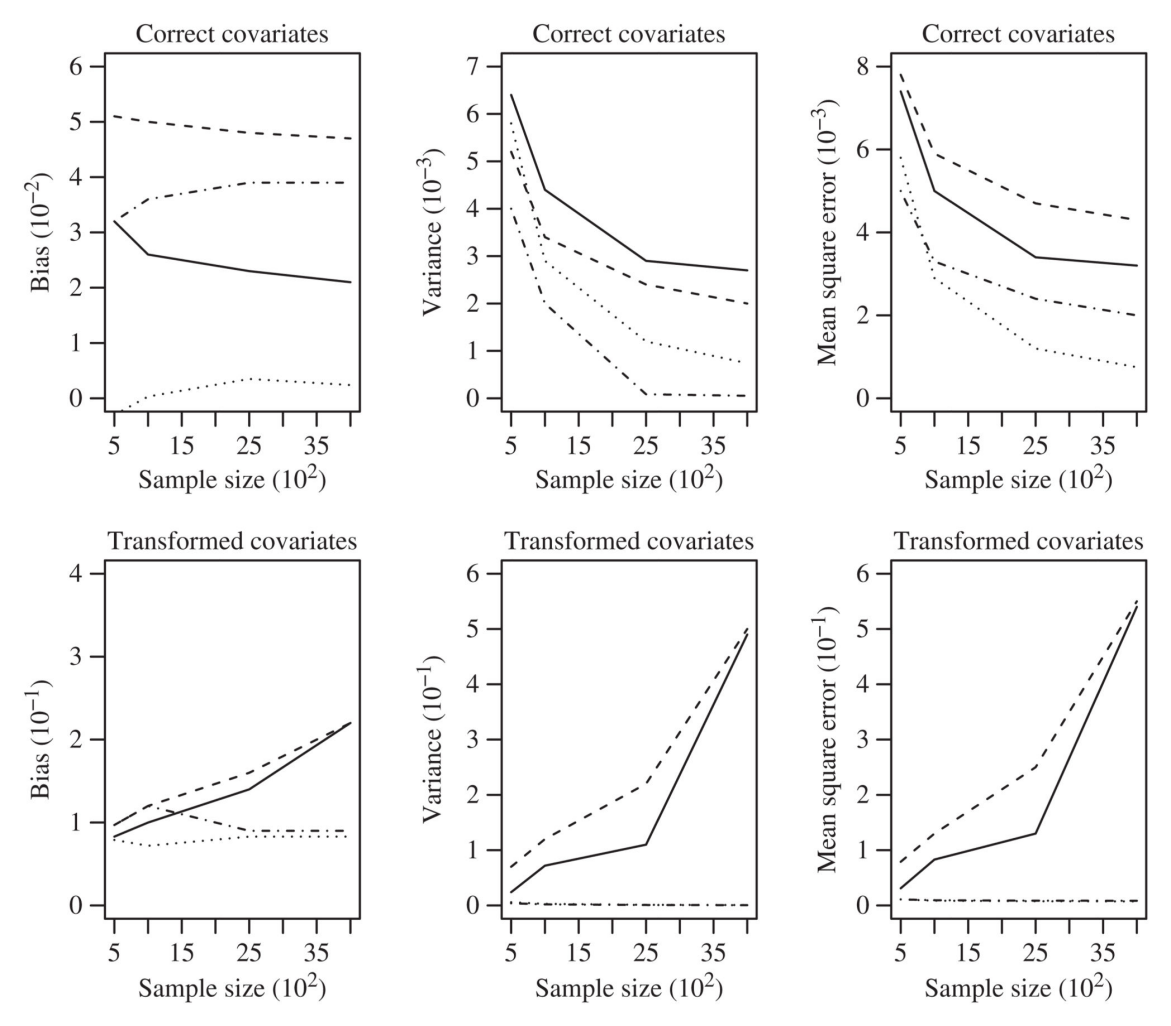
Plots of the bias (left panels), empirical variance (middle panels) and mean squared error (right panels) of the treatment effect estimates as a function of sample size, for the correct (top panels) and transformed (bottom panels) covariate sets; in each panel the different line types represent Approach 1 with model structure A (dotted), Approach 1 with model structure B (dot-dash), Approach 2 with model structure A (solid), and Approach 2 with model structure B (dashed).

The left panels of Fig. 1 show that the estimates from Approach 1 have smaller biases than those from Approach 2, particularly when the covariates are transformed. In this case, bias increases with sample size in Approach 2 but not in Approach 1. When the correct model structure A is used with the correct covariates, Approach 1 has smaller bias than Approach 2, perhaps because Approach 2 requires the law of large numbers to work well before the associations between covariates and treatment assignment can be eliminated after weighting.

The middle panels of Fig. 1 present the empirical variances of the treatment effect estimates. When the correct covariates are used, both approaches have small variances that decrease with sample size, although Approach 1 is better. Within each approach, estimates from the incorrect model structure B are less variable than those from model structure A, perhaps because the weights are less variable under model structure B as it has fewer degrees of freedom. The variances under Approach 1, but not under Approach 2, decrease with sample size when the transformed covariates are used. This behaviour in Approach 2 with the transformed covariates is due to a few sets of estimated weights exacerbating the extremeness of the tails of the sampling distribution as the sample size increases; see Robins et al. (2007, pp. 553–4) for more details. Similar phenomena are observed with the mean squared error.

Because Approach 2 performs so poorly relative to Approach 1 when the transformed covariates are used, it is difficult to distinguish between the performances of Approach 1 under model structures A and B in Fig. 1, so we summarize the results here: within Approach 1, estimates from model structure A have smaller biases but larger variances than estimates from model structure B. Overall, estimates from model structure A have smaller mean squared errors for *n* ≥ 1000.

In summary, in all examined scenarios, estimates based on the proposed method have smaller biases and variances than the maximum likelihood estimates.

## 7 Application

Steroids are effective and low-cost treatments for relieving disease activity in patients with systemic lupus erythematosus, a chronic autoimmune disease that affects multiple organ systems. However, there is evidence that steroid exposure could be associated with permanent organ damage that might not be attributable to disease activity. Motivated by a recent study (Bruce et al., 2015), we aim to estimate the causal dose-response relationship between steroid exposure and damage accrual shortly after disease diagnosis using data from the Systemic Lupus International Collaborating Clinics inception cohort.

We focus on 1342 patients who were enrolled between 1999 and 2011 from 32 sites and had at least two yearly assessments in damage accrual after disease diagnosis. The outcome of interest *Y_i_* is the number of items accrued in the Systemic Lupus International Collaborating Clinics/American College of Rheumatology Damage Index during the period *O_i_*, defined as the time interval between the two initial assessments in years. The semicontinuous treatment variable *T_i_* is the average steroid dose per day in milligrams over the same time period. Based on clinical inputs, we consider the following pretreatment covariates *X_i_*: the numerical scoring version of the British Isles Lupus Assessment Group disease activity index (Hay et al., 1993; Ehrenstein et al., 1995), which was developed according to the principle of physicians’ intentions to treat; age at diagnosis in years; disease duration in years; and the groups of race/ethnicity and geographic region.

We consider the model (11) for *T_i_* with *g*(*x*) = log(*x* + 1) to reduce right skewness in the positive steroid dose. For ${\tilde{X}}_{i}$, we include the main effects of *X_i_*. We use the same four models as in the simulations to estimate weights. Further details can be found in the Supplementary Material.

We model *Y_i_* with a weighted negative binomial model (14), where for examining the effect of presence of steroid use on damage accrual we specify *λ_i_* = *E*(*Y_i_* | *T_i_*; *γ*_0_, *γ*_1_) = *O_i_* exp{*γ*_0_ + *γ*_1_*I*(*T_i_* > 0)}, and for examining the dose-response relationship we specify *λ_i_* = *E*(*Y_i_* | *T_i_*; *ξ*_0_, *ξ*_1_) = *O_i_* exp{*ξ*_0_ + *ξ*_1_ log(*T_i_* + 1)}, with *γ*_0_, *γ*_1_, *ξ*_0_, *ξ*_1_ and *θ* parameters to be estimated. We construct 95% bootstrap percentile confidence intervals for parameter estimates using 1000 nonparametric bootstrap samples.

The results of the outcome regression models based on four sets of estimated weights are reported in Table 1. Consistent with current clinical evidence (Bruce et al., 2015), estimates from all weighted outcome regression models indicate that steroid use and the average steroid dose are positively and significantly associated with damage accrual in the initial period following diagnosis, although the estimated effect sizes from Approach 1 are larger than those from Approach 2. Approach 1 also yields narrower confidence intervals than Approach 2. Within each approach, model structure B gives slightly smaller standard errors than model structure A.

We also fitted models with *I* (*T_i_* > 0) and *I* (*T_i_* > 0) log(*T_i_* + 1) included in the outcome regression. This provides the dose-response relationship after removing the patients unexposed to steroids (Greenland & Poole, 1995). The estimated dose-response functions were similar to those obtained from the models that include the semicontinuous treatment.

Overall, our results suggest that steroid dose is related to the damage accrual rate at the early disease stage of systemic lupus erythematosus. This suggests that clinicians might need to seek steroid-sparing therapies even at the early disease stage in order to reduce damage accrual.

## 8 Longitudinal setting

Our framework can be extended to the longitudinal setting. Here we give an example using a similar setting to that in Moodie & Stephens (2010). Suppose that for the *i*th unit (*i* = 1, … , *n*), in each time interval [*s*_*ij*−1_, *s_ij_*) (*j* = 1, … , *m_i_*; *s*_*i*0_ = 0) of a longitudinal study, we observe in chronological order a vector of covariates *X_ij_*, a time-varying treatment *T_ij_* that can be of any distribution, and a longitudinal outcome *Y_ij_*. The units are not necessarily followed up at the same time-points. Let the random variable $Y_{ij}\left(t_{ij}^{\text{P}}\right)$ be the potential outcome that would have arisen had treatment $t_{ij}^{\text{P}}$ been administered in the time interval [*s*_*ij*−1_, *s_ij_*). We consider the setting where interest lies in estimating the direct causal effect of $t_{ij}^{\text{P}}$ on $E\left\{Y_{ij}\left(t_{ij}^{\text{P}}\right)\right\},$ which may be confounded by histories of covariates, treatment assignment and response, ${\bar{X}}_{ij},{\bar{T}}_{ij-1}$ and *Ȳ*_*ij*−1_. Here an overbar represents the history of a process; for example, ${\bar{X}}_{ij}=(X_{i1},\ldots ,X_{ij}).$

In order to identify the direct causal effect of $t_{ij}^{\text{P}}$ on $E\left\{Y_{ij}\left(t_{ij}^{\text{P}}\right)\right\},$ we make the sequential ignorability assumption, $\text{pr}\{T_{ij}\;|\;Y_{ij}\left(t_{ij}^{\text{P}}\right),{\bar{X}}_{ij},{\bar{T}}_{ij-1},{\bar{Y}}_{ij-1}\}=\text{pr}\left(T_{ij}|{\bar{X}}_{ij},{\bar{T}}_{ij-1},{\bar{Y}}_{ij-1}\right)$ for any time interval, and the positivity assumption, $\text{pr}\left(T_{ij}\in A\;|\;{\bar{X}}_{ij},{\bar{T}}_{ij-1},{\bar{Y}}_{ij-1}\right)>0$ for all ${\bar{X}}_{ij},{\bar{T}}_{ij-1}$ and *Ȳ*_*ij*−1_ and for any set 𝒜 with positive measure. Under these assumptions, the effect of $t_{ij}^{\text{P}}$ on $E\left\{Y_{ij}\left(t_{ij}^{\text{P}}\right)\right\}$ can be consistently estimated by weighting the *i*th unit’s data in the interval [*s*_*ij*−1_, *s*_*ij*_) with $W_{ij}=\text{pr}\left(T_{ij}\right)/\text{pr}\left(T_{ij}\;|\;{\bar{X}}_{ij},{\bar{T}}_{ij-1},{\bar{Y}}_{ij-1}\right)$ for all units and time intervals. The weights are typically estimated by $W_{ij}=\text{pr}\left(T_{ij};\hat{\alpha }\right)/\text{pr}\left(T_{ij}\;|\;{\tilde{X}}_{ij};\hat{\beta }\right),$ where ${\tilde{X}}_{ij}$ are functionals of ${\bar{X}}_{ij},{\bar{T}}_{ij-1}$ and *Ȳ*_*ij*−1_, and $\hat{\alpha }$ and $\hat{\beta }$ are the maximum likelihood estimates of *α* and *β*.

An alternative approach to weight estimation is to generalize our proposed method. Following the same strategy as in § 3·1, we assume that given known weights *W_ij_*, the time-varying propensity function in the time interval [*s*_*ij*−1_, *s_ij_*) follows a model $\text{pr}\{T_{ij}\;|\;{\tilde{X}}_{ij};\beta \left(W\right)\}.$ As in § 3·1, we partition *β*(*W*) into {*β*_b_(*W*), *β*_d_ (*W*)}, where *β*_d_ (*W*) are regression coefficients that characterize the association between ${\tilde{X}}_{ij}$ and *T_ij_* over time, and *β*_b_(*W*) are parameters that characterize the baseline distribution, e.g., the intercept terms. Similarly to before, conditions for weight estimation can be derived by inverting the weighted score equations

(15) $$\sum_{i=1}^{n}\sum_{j=1}^{m_{i}}W_{ij}\frac{\partial }{\partial \beta (W)}\text{log}\;\text{pr}\left\{T_{ij}\;|\;{\tilde{X}}_{ij};\beta (W)\right\}{|}_{\left\{\beta_{\text{b}}(W)=\hat{\alpha },\beta_{\text{d}}(W)=0\right\}}=0,$$

where $\hat{\alpha }$ is obtained by fitting the model pr(*T_ij_*; *α*) to the observed data for the time-varying treatment *T_ij_*. Thus these conditions are designed to eliminate the association between ${\tilde{X}}_{ij}$ and *T_ij_* and to preserve the observed marginal distribution of *T_ij_* after weighting. Other projection functions that can help to further stabilize the weights, such as those that depend on some of the covariates, can also be considered in the proposed framework with only minor modifications. This would be useful when the interactions between these covariates and treatment are of interest in the outcome regression model. Finally, the convex optimization approach in § 3·2 can be used for weight estimation by replacing the equations (4) with (15). For large sample sizes, a parametric approach to solving the conditions (15) would be useful.

## 9 Discussion

The proposed method has some limitations. First, both our method and existing covariate balancing weight methods treat covariates equally and balance them simultaneously. This can lead to poor performance in high-dimensional settings, so it would be of interest to incorporate different weights for the covariates when estimating the weights for the units. Second, it can be hard to detect near violations of the positivity assumption with our method, because it generally results in small variance of the causal effect estimates by exactly balancing the covariates. In such circumstances, e.g., when there is strong confounding, the results from our method can hide the fact that the observed data alone carry little information about the target causal effect and can have large bias under model misspecification because our method will almost entirely rely on extrapolation. It is therefore important to assess the positivity assumption when applying our framework. Third, our method does not necessarily estimate the causal effect for the population of interest, such as the target population of a health policy. This can be remedied by supplementing the conditions (4) with the additional conditions $\sum_{i=1}^{n}W_{i}{\tilde{X}}_{i}/n=c,$ where *c* is the sample mean of ${\tilde{X}}_{i}$ with respect to the target population (Stuart et al., 2011).

## Supplementary Material

Supplementary material available at *Biometrika* online includes properties of stabilized inverse probability of treatment weights, derivations of conditions for other treatment distributions, simulation results and details of the application.

Supplementary information

## Figures and Tables

**Table 1:** Parameter estimates and 95% confidence intervals from fitting the weighted outcome regression models to the systemic lupus erythematosus data

	Approach 1		Approach 2	
	Model structure A	Model structure B	Model structure A	Model structure B
Binary treatment				
*γ*_0_	−2·50 (−2·93, −2·19)	−2·50 (−2·93, −2·18)	−2·36 (−2·87, −1·98)	−2·36 (−·87, −1·98)
*γ*_1_	0·76 (0·41, 1·21)	0·73 (0·37, 1·19)	0·57 (0·10, 1·10)	0·57 (0·12, 1·10)
*θ*	1·60 (0·77, 2·53)	1·62 (0·83, 2·54)	1·30 (0·38, 2·48)	1·19 (0·23, 2·41)
Semicontinuous treatment				
*ξ*_0_	−2·57 (−2·93, −2·29)	−2·54 (−2·88, −2·27)	−2·47 (−2·89, −2·11)	−2·45 (−2·84, −2·12)
*ξ*_1_	0·40 (0·24, 0·56)	0·37 (0·23, 0·52)	0·33 (0·16, 0·52)	0·32 (0·15, 0·49)
*θ*	1·41 (0·70, 2·27)	1·48 (0·75, 2·32)	1·17 (0·34, 2·22)	1·11 (0·28, 2·14)
