# Supplementary material for: Covariate association eliminating weights: a unified weighting framework for causal effect estimation
Source: Biometrika. Author manuscript; Available in PMC 2019 Apr 24. (PMC6481550; doi:10.1093/biomet/asy015)
Supplement: Supplementary information [file NIHMS82525-supplement-Supplementary_information.pdf]

Biometrika (2018), **xx**, x, *pp.* 1–8
© 2007 Biometrika Trust
Printed in Great Britain

Supplementary Material for "Covariate association eliminating weights: a unified weighting framework for causal effect estimation"

BY SEAN YIU AND LI SU

Medical Research Council Biostatistics Unit, School of Clinical Medicine, University of Cambridge, Robinson Way, Cambridge, CB2 0SR, U.K.

sean.yiu@mrc-bsu.cam.ac.uk li.su@mrc-bsu.cam.ac.uk

1. PROPERTIES OF STABILIZED INVERSE PROBABILITY OF TREATMENT WEIGHTS

We show that $\text{pr}^*(T_i \mid X_i) = \text{pr}(T_i)$ and $\text{pr}^*(Y_i \mid T_i, X_i) = \text{pr}(Y_i \mid T_i, X_i)$ if $W_i = W_i(T_i, X_i) = \text{pr}(T_i)/\text{pr}(T_i \mid X_i)$. Without loss of generality, suppose T_i and Y_i are continuous on the real line. Then

$$\text{pr}^*(T_i \mid X_i) = \frac{\text{pr}(T_i \mid X_i)W_i(T_i, X_i)}{\int_{-\infty}^{\infty} \text{pr}(T_i \mid X_i)W_i(T_i, X_i)\, dT_i} = \frac{\text{pr}(T_i)}{\int_{-\infty}^{\infty} \text{pr}(T_i)\, dT_i} = \text{pr}(T_i),$$

$$\text{pr}^*(Y_i \mid T_i, X_i) = \frac{\text{pr}(Y_i \mid T_i, X_i)W_i(T_i, X_i)}{\int_{-\infty}^{\infty} \text{pr}(Y_i \mid T_i, X_i)W_i(T_i, X_i)\, dY_i} = \frac{\text{pr}(Y_i \mid T_i, X_i)}{\int_{-\infty}^{\infty} \text{pr}(Y_i \mid T_i, X_i)\, dY_i} = \text{pr}(Y_i \mid T_i, X_i).$$

2. CATEGORICAL TREATMENT

The covariate balancing conditions for categorical treatments with J categories are (Imai & Ratkovic, 2015)

$$\frac{1}{n}\sum_{i=1}^{n} X_i^* = \frac{1}{n}\sum_{i=1}^{n} I(T_i = j)W_i X_i^* \;(j = 1, \ldots, J), \tag{1}$$

where $X_i^* = (1, \tilde{X}_i^{\text{T}})^{\text{T}}$ and $I(\cdot)$ is an indicator function. For identifiability one set of conditions is redundant and so there are $J - 1$ sets of conditions in total. Because X_i^* includes 1, these conditions constrain the number of units in each category to be equal to n in the weighted data. The other conditions constrain the weighted mean of $\tilde{X}_i$ in each category to be equal to the overall mean of $\tilde{X}_i$ in the observed data. Using the proposed framework, we specify a multinomial logistic regression model for the propensity function in the weighted data

$$\text{pr}\{T_i = j \mid \tilde{X}_i; \beta(W)\} = \frac{\exp\{\beta_j(W)^{\text{T}} X_i^*\}}{1 + \sum_{j=1}^{J-1} \exp\{\beta_j(W)^{\text{T}} X_i^*\}}$$

with $\beta_J(W) = 0$. The corresponding score equations are

$$\sum_{i=1}^{n} W_i X_i^* \left[I(T_i = j) - \frac{\exp\{\beta_j(W)^{\text{T}} X_i^*\}}{1 + \sum_{j=1}^{J-1} \exp\{\beta_j(W)^{\text{T}} X_i^*\}} \right] = 0.$$

The next step is to force the regression coefficients for $\tilde{X}_i$ to zeros, and the intercept terms in $\beta(W)$ to preserve the marginal distribution of T_i. This leads to the set of conditions

$$\sum_{i=1}^{n} W_i X_i^* \left\{ I(T_i = j) - \hat{\pi}_j \right\} = 0, \tag{2}$$

where $\hat{\pi}_j$ is the observed proportion of units in the treatment category j. This set of conditions impose equality in the numbers of units in the weighted and observed data for each treatment category, since X_i^* includes 1. They also constrain the mean of $\tilde{X}_i$ in each category in the weighted data to be equal to the overall weighted mean of $\tilde{X}_i$. Hence (2) is analogous to (1).

3. TWO-PART LOG-SKEW-NORMAL MODEL

Another approach to modelling semi-continuous treatments is to specify a model that can account for right skewness in the distribution of the positive values of T_i, such as the flexible two-part log-skew-normal model in Smith et al. (2014)

$$\mathrm{pr}(T_i \mid \tilde{X}_i; \pi_i, \omega_i, \xi_i, \kappa) = (1-\pi_i)^{1-\delta_i} \left[\frac{2\pi_i}{\omega_i T_i} \phi\left(\frac{\log T_i - \xi_i}{\omega_i} \right) \Phi\left\{ \kappa \left(\frac{\log T_i - \xi_i}{\omega_i} \right) \right\} \right]^{\delta_i}, \tag{3}$$

where $\pi_i = 1/[1 + \exp\{-\beta_\pi(W)^{\mathrm{T}} X_i^*\}]$, $\xi_i = \beta_\xi(W)^{\mathrm{T}} X_i^*$, $\omega_i = \exp\{\beta_\omega(W)^{\mathrm{T}} X_i^*\}$, $\kappa = \kappa(W)$, $X_i^* = (1, \tilde{X}_i^{\mathrm{T}})^{\mathrm{T}}$, $\delta_i = I(T_i > 0)$, where $I(\cdot)$ is an indicator function, and $\phi(\cdot)$ and $\Phi(\cdot)$ are standard normal density and distribution functions. The parameter κ accounts for skewness in the positive values of T_i; a positive/negative value of κ indicates that the distribution is right/left skewed, while $\kappa = 0$ corresponds to the two-part log-normal distribution. The weighted score equations for the parameters in the continuous component of the model are

$$\sum_{i=1}^{n} W_i X_i^* \delta_i \left[\left(\frac{\log T_i - \xi_i}{\omega_i^2} \right) - \frac{\kappa}{\omega_i} \frac{\phi\{\kappa(\log T_i - \xi_i)/\omega_i\}}{\Phi\{\kappa(\log T_i - \xi_i)/\omega_i\}} \right] = 0, \tag{4}$$

$$\sum_{i=1}^{n} W_i X_i^* \delta_i \left[-1 + \left(\frac{\log T_i - \xi_i}{\omega_i} \right)^2 - \frac{\kappa(\log T_i - \xi_i)}{\omega_i} \frac{\phi\{\kappa(\log T_i - \xi_i)/\omega_i\}}{\Phi\{\kappa(\log T_i - \xi_i)/\omega_i\}} \right] = 0, \tag{5}$$

$$\sum_{i=1}^{n} W_i \delta_i \left[\left(\frac{\log T_i - \xi_i}{\omega_i} \right) \frac{\phi\{\kappa(\log T_i - \xi_i)/\omega_i\}}{\Phi\{\kappa(\log T_i - \xi_i)/\omega_i\}} \right] = 0, \tag{6}$$

where (4)–(6) corresponds to the score equations for $\beta_\xi(W)$, $\beta_\omega(W)$ and $\kappa(W)$, respectively. Together with the conditions from the binary component, one set of conditions are

$$\sum_{i=1}^{n} W_i X_i^* \left\{ \delta_i - \hat{\pi}_0 \right\} = 0, \quad \sum_{i=1}^{n} W_i X_i^* \delta_i \left(\frac{\log T_i - \hat{\xi}_0}{\hat{\omega}_0^2} \right) = 0,$$

$$\sum_{i=1}^{n} W_i X_i^* \delta_i \left\{ -1 + \frac{(\log T_i - \hat{\xi}_0)^2}{\hat{\omega}_0^2} \right\} = 0, \quad \sum_{i=1}^{n} W_i \delta_i \left(\frac{\log T_i - \hat{\xi}_0}{\hat{\omega}_0} \right) = 0,$$

where $\hat{\pi}_0$, $\hat{\xi}_0$ and $\hat{\omega}_0$ are the maximum likelihood estimates of π_i, ξ_i and ω_i obtained by fitting (3), but without covariates and with $\kappa = 0$, to the observed treatment data. Fixing $\kappa = 0$ in the projection function does not invalidate the constructed weights for consistently estimating causal treatment effects.

4. Count treatment

In many situations it is of interest to investigate the causal dose-response relationship between a count treatment and the outcome. A common example is the effect of treatment frequency, for instance, the effect of smoking frequency on annual medical expenditure (Imai & van Dyk, 2004) or the effect of the number of prescribed pulse steroids, a common treatment in systemic lupus erythematosus, on organ damage accrual (Mosca et al., 2011).

A natural approach to modelling a count treatment T_i is to specify a negative binomial model

$$\mathrm{pr}(T_i \mid \tilde{X}_i; \theta_i, \mu_i) = \frac{\Gamma(T_i + 1/\theta_i)}{\Gamma(1/\theta_i) T_i!} \left(\frac{\theta_i \mu_i}{1 + \theta_i \mu_i} \right)^{T_i} \left(\frac{1}{1 + \theta_i \mu_i} \right)^{1/\theta_i} \quad (T_i = 0, 1, \ldots), \qquad (7)$$

where $\mu_i = E\{T_i \mid \tilde{X}_i; \beta_\mu(W)\} = \exp\{\beta_\mu(W)^{\mathrm{T}} X_i^*\}$, $\theta_i = \exp\{\beta_\theta(W)^{\mathrm{T}} X_i^*\}$ and $X_i^* = (1, \tilde{X}_i^{\mathrm{T}})^{\mathrm{T}}$. The parameter θ_i accounts for over-dispersion, a common phenomenon in many count data in practice. Specifically, $\mathrm{var}\{T_i \mid \tilde{X}_i; \beta_\mu(W), \beta_\theta(W)\} = \mu_i(1 + \theta_i \mu_i)$. The corresponding score equations for the parameters $\beta_\mu(W)$ and $\beta_\theta(W)$ are

$$\sum_{i=1}^n W_i X_i^* \left(\frac{T_i - \mu_i}{1 + \theta_i \mu_i} \right) = 0, \qquad (8)$$

$$\sum_{i=1}^n W_i \frac{X_i^*}{\theta_i} \left\{ \frac{\theta_i (T_i - \mu_i)}{1 + \theta_i \mu_i} + \log(1 + \theta_i \mu_i) - \psi(T_i + 1/\theta_i) + \psi(1/\theta_i) \right\} = 0, \qquad (9)$$

where $\psi(x)$ is the derivative of the $\log \Gamma(x)$ function. In order for $\tilde{X}_i$ and T_i to be unassociated as described by (7) in the weighted data, we find weights W as solutions to the conditions

$$\sum_{i=1}^n W_i X_i^* \left(\frac{T_i - \hat{\mu}_0}{1 + \hat{\theta}_0 \hat{\mu}_0} \right) = 0, \qquad (10)$$

$$\sum_{i=1}^n W_i \frac{X_i^*}{\hat{\theta}_0} \left\{ \frac{\hat{\theta}_0 (T_i - \hat{\mu}_0)}{1 + \hat{\theta}_0 \hat{\mu}_0} + \log(1 + \hat{\theta}_0 \hat{\mu}_0) - \psi(T_i + 1/\hat{\theta}_0) + \psi(1/\hat{\theta}_0) \right\} = 0, \qquad (11)$$

where $\hat{\mu}_0$ and $\hat{\theta}_0$ are obtained by fitting (7), but without covariates, to the observed treatment data. Conditions (10) and (11) are obtained by fixing the regression coefficient components of $\beta_\mu(W)$ and $\beta_\theta(W)$ to zeros and the exponential of the intercept terms to $\hat{\mu}_0$ and $\hat{\theta}_0$ in (8) and (9).

5. Additional simulation results

Because Approach 2 performs so poorly relative to Approach 1 when the transformed covariates are used, it is difficult to distinguish between the performance of Approach 1 under model structures A and B in Fig. 1 of the main text. We therefore summarize the corresponding results in Table 1. Within Approach 1, estimates from model structure A have smaller biases but larger variances than estimates from model structure B. Overall, estimates from model structure A have smaller mean square errors for $n \geq 1000$.

6. Further Details of the Application

6·1. *Descriptive statistics*

The mean of the time period O_i was 1.36 years with standard deviation 0.87 years. Of the 1342 patients, 1156 patients had no damage accrual within O_i, while the remaining 186 patients

Table 1: *Simulation results from Approach 1 under model structures A and B with transformed covariates*

	Model structure A			Model structure B		
	Bias (10^{-2})	Variance (10^{-3})	Mean square error (10^{-2})	Bias (10^{-2})	Variance (10^{-3})	Mean square error (10^{-2})
$n = 500$	7.9	4.30	1.10	8.6	3.30	1.10
$n = 1000$	7.2	2.10	0.87	12.0	1.60	0.95
$n = 2500$	8.3	0.87	0.77	9.0	0.66	0.87
$n = 4000$	8.3	0.54	0.74	9.0	0.41	0.85

developed between one and four damaged items. Steroids were prescribed to 907 patients. The mean positive steroid dose was 9.04 milligrams per day with standard deviation 8.23 milligrams per day. The mean British Isles Lupus Assessment Group disease activity index was 0.51 with standard deviation 0.49. The mean age at diagnosis was 34.31 years with standard deviation 13.55 years. The mean disease duration was 1.43 years with standard deviation 0.91 years. The race/geographic region groups comprised of 118 Caucasians from the United States, 171 Hispanics from Mexico, 31 Hispanics from elsewhere, 65 Africans from the United States, 138 Africans from elsewhere, 229 Asians, 46 patients with other ethnicities and 544 Caucasians from Canada and Europe, which was the reference group.

6·2. *Details of the estimated weights*

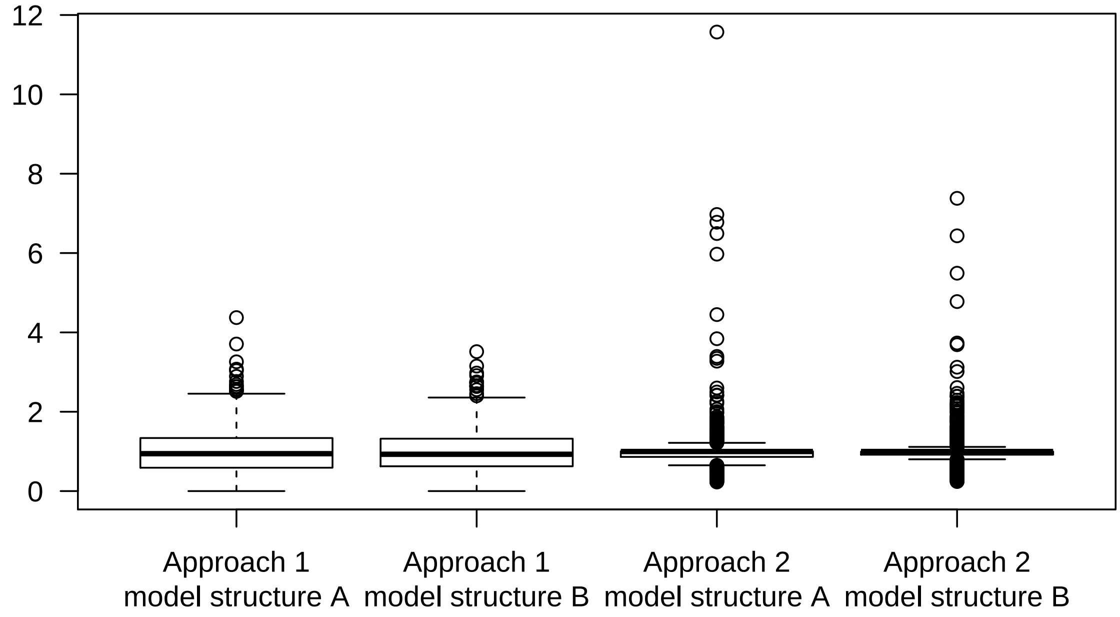

Fig. 1: Box plots of the estimated weights from the application in § 7 of the main text after scaling by their averages.

Figure 1 shows that the weights from Approach 2 have larger ranges than those from Approach 1. Furthermore, several weights from Approach 2 are greater than the largest weight from Approach 1, which is 4.37. The variances of the weights from Approaches 1 and 2 are 0.32 and 0.27, and 0.53 and 0.43 under model structures A and B. Thus model structure B gives less variable weights, which was also seen in the simulations. For Approach 1, 42 and 36 weights are zeros under model structures A and B. Interestingly, these weights correspond to small weights under Approach 2; they all practically lie within the first decile, implying that the observed treatments for the respective patients had large estimated conditional probabilities of occurrence. This suggests that the proposed method is removing a subset of units that have most of their estimated propensity function mass concentrated on a small interval around their observed treatment, i.e., those that are close to, or are in fact, violating the positivity assumption that was stated in § 2 of the main text; see Peterson et al. (2010) for a comprehensive discussion. This is similar to removing units with extreme propensity scores (Crump et al., 2009).

Apart from examining the stability of estimated weights, researchers are also encouraged to check covariate balance before estimating causal treatment effects. A natural approach to assessing covariate balance is to apply weighted regression to the observed treatment data and then to use standard statistical tests and confidence intervals to examine covariate associations. We follow this approach by fitting (11) of the main text to the data weighted by the weights from Approach 2. Unlike Approach 1, Approach 2 does not necessarily balance covariates. We construct 95% percentile confidence intervals with 1000 non-parametric bootstrap samples. For comparison, we apply the same procedure to the observed data.

Table 2 shows that many covariates are strongly associated with treatment assignment in the observed data. For example, British Isles Lupus Assessment Group disease activity index, as the measure of disease activity, is positively associated with steroid prescription and steroid dose. Reassuringly, both models under Approach 2 have greatly reduced most of the associations between covariates and treatment assignment, as indicated by smaller estimated regression coefficients. An interesting exception occurs for other ethnicities, which appears to have a stronger association with the variance of steroid dose in the weighted data. Most of the confidence intervals also suggest that there is insufficient evidence to reject the hypothesis of reasonable covariate balance. However, following Imai et al. (2008), caution should be applied when interpreting estimated confidence intervals with regard to covariate balance since wide intervals may be more indicative of a lack of statistical power to detect imbalances of observed covariates, e.g., due to highly variable weights, rather than an improvement in covariate balance. More importantly, estimated confidence intervals should not be used as stopping rules for improving covariate balance because an arbitrarily small non-zero regression coefficient estimate, even if it is within a narrow confidence interval containing zero, can still lead to substantial bias in treatment effect estimates (Imai et al., 2008). Our method aligns well with this caution as it forces covariates to be balanced across treatment levels as specified by a chosen propensity function model.

As an additional diagnostic check for covariate balance, we calculate the standardized mean differences in covariates within quintiles of the positive steroid dose $(0, 1.13]$, $(1.13, 3.61]$, $(3.61, 7.34]$, $(7.34, 13.75]$ and > 13.75 milligrams per day relative to no steroids, in the observed and weighted data. Table 3 presents the results for both approaches under model structure A, the results for model structure B are similar. From Table 3, it is clear that the standardized mean differences in the observed data are relatively large, particularly for an important confounder, British Isles Lupus Assessment Group disease activity index, in the > 13.75 milligrams per day category. In contrast, both weighting approaches greatly reduce the standardized mean differences relative to the observed data, though Approach 1 is generally better, especially for the strong confounder British Isles Lupus Assessment Group disease activity index.

Table 2: *Parameter estimates and 95% confidence intervals from fitting (11) of the main text to the observed and weighted data, with weights from Approach 2*

	Unweighted	Model structure A	Model structure B
Binary component:			
Intercept	−0.11 (−0.42, 0.17)	0.81 (0.60, 0.99)	0.83 (0.61, 1.01)
BILAG	1.05 (0.72, 1.40)	−0.05 (−0.30, 0.36)	−0.06 (−0.30, 0.34)
Age at diagnosis*	−0.16 (−0.29, −0.03)	−0.07 (−0.17, 0.01)	−0.08 (−0.16, 0.00)
Disease duration	−0.14 (−0.26, 0.01)	−0.03 (−0.11, 0.03)	−0.03 (−0.10, 0.03)
Caucasian/USA	−0.01 (−0.42, 0.43)	0.09 (−0.16, 0.33)	0.10 (−0.17, 0.39)
Hispanic/Mexico	1.39 (0.97, 1.90)	0.10 (−0.21, 0.40)	0.09 (−0.23, 0.37)
Hispanic/elsewhere	0.14 (−0.66, 0.96)	−0.01 (−0.21, 0.16)	0.12 (−0.18, 0.55)
African/USA	1.47 (0.84, 2.30)	−0.07 (−0.44, 0.27)	−0.04 (−0.41, 0.31)
African/elsewhere	1.25 (0.80, 1.79)	−0.05 (−0.21, 0.11)	−0.06 (−0.23, 0.09)
Asian	1.74 (1.32, 2.19)	0.15 (−0.04, 0.36)	0.08 (−0.09, 0.23)
Other ethnicities	0.42 (−0.25, 1.17)	−0.11 (−0.26, 0.13)	−0.08 (−0.24, 0.05)
Continuous component: mean model			
Intercept	1.78 (1.64, 1.91)	1.92 (1.79, 2.07)	1.94 (1.84, 2.06)
BILAG	0.39 (0.30, 0.49)	−0.02 (−0.16, 0.10)	0.02 (−0.07, 0.12)
Age at diagnosis*	−0.04 (−0.09, 0.02)	0.04 (−0.02, 0.10)	0.02 (−0.02, 0.05)
Disease duration	−0.03 (−0.08, 0.03)	0.03 (−0.06, 0.10)	0.01 (−0.05, 0.05)
Caucasian/USA	−0.39 (−0.64, −0.13)	0.10 (−0.17, 0.37)	0.22 (−0.17, 0.60)
Hispanic/Mexico	0.17 (−0.01, 0.33)	0.09 (−0.03, 0.19)	0.04 (−0.06, 0.13)
Hispanic/elsewhere	0.36 (−0.09, 0.77)	0.07 (−0.15, 0.31)	0.00 (−0.71, 0.48)
African/USA	−0.09 (−0.36, 0.16)	0.08 (−0.03, 0.22)	0.16 (−0.01, 0.43)
African/elsewhere	0.20 (0.05, 0.34)	0.01 (−0.15, 0.13)	0.06 (−0.03, 0.14)
Asian	0.03 (−0.10, 0.16)	0.03 (−0.20, 0.24)	0.02 (−0.07, 0.09)
Other ethnicities	0.20 (−0.13, 0.49)	0.04 (−0.38, 0.19)	−0.05 (−0.24, 0.13)
standard deviation model			
Intercept	−0.01 (−0.25, 0.02)	−0.24 (−0.34, −0.11)	−0.08 (−0.23, 0.08)
BILAG	−0.04 (−0.14, 0.05)	0.03 (−0.13, 0.18)	0.00 (−0.15, 0.15)
Age at diagnosis*	−0.04 (−0.10, 0.02)	−0.02 (−0.08, 0.01)	0.07 (−0.14, −0.01)
Disease duration	−0.09 (−0.16, −0.02)	−0.02 (−0.09, 0.03)	−0.09 (−0.18, −0.02)
Caucasian/USA	0.13 (−0.06, 0.29)	0.09 (−0.12, 0.22)	0.16 (−0.15, 0.32)
Hispanic/Mexico	0.02 (−0.14, 0.16)	0.02 (−0.08, 0.12)	0.01 (−0.16, 0.17)
Hispanic/elsewhere	0.17 (−0.36, 0.45)	0.03 (−0.24, 0.21)	0.25 (−0.38, 0.49)
African/USA	0.12 (−0.10, 0.30)	0.06 (−0.06, 0.16)	0.16 (−0.11, 0.33)
African/elsewhere	−0.16 (−0.34, 0.00)	0.05 (−0.07, 0.17)	−0.11 (−0.31, 0.05)
Asian	−0.26 (−0.40, −0.12)	0.04 (−0.11, 0.13)	−0.25 (−0.41, −0.09)
Other ethnicities	0.02 (−0.30, 0.26)	0.16 (0.017, 0.31)	0.15 (−0.12, 0.35)

*, standardized version of the covariate; BILAG, British Isles Lupus Assessment Group disease activity index; USA, United States of America.

Table 3: *Standardized mean differences in covariates within quintiles of positive steroid dose relative to no steroids in the observed and weighted data under model structure A*

	Steroid dose categories				
	$(0, 1.1.3]$	$(1.13, 3.61]$	$(3.61, 7.34]$	$(7.34, 13.75]$	> 13.75
BILAG	(10^{-2})	(10^{-2})	(10^{-2})	(10^{-2})	(10^{-2})
Unweighted	2.13	9.23	18.97	35.00	63.20
Approach 1	2.14	0.70	−2.80	0.42	2.01
Approach 2	4.83	−6.38	−1.88	−4.42	1.67
*Age at diagnosis**					
Unweighted	−12.00	−29.68	−18.81	−19.86	−41.33
Approach 1	7.51	−14.63	0.17	4.05	−0.74
Approach 2	−11.51	−21.20	−3.51	−0.94	−3.42
Disease duration					
Unweighted	−13.40	−3.25	−5.21	−9.60	−10.07
Approach 1	−0.48	5.29	−3.12	−2.86	5.30
Approach 2	−8.59	2.09	−2.47	−5.32	6.42
Caucasian/USA					
Unweighted	10.00	−4.18	−18.89	−25.54	−23.67
Approach 1	−0.48	2.72	1.95	−3.32	0.39
Approach 2	−1.54	4.55	−0.69	−2.79	6.61
Hispanic/Mexico					
Unweighted	5.89	22.10	19.48	15.90	35.80
Approach 1	−7.17	6.41	2.70	−3.97	0.52
Approach 2	−7.31	8.74	4.51	−1.84	5.52
Hispanic/elsewhere					
Unweighted	−8.62	1.07	−14.45	−1.26	−0.48
Approach 1	−6.17	11.65	−10.00	3.35	−2.18
Approach 2	−5.44	10.35	−9.72	3.27	−1.32
African/USA					
Unweighted	26.79	8.79	14.08	0.72	16.10
Approach 1	0.01	−3.90	6.36	−7.03	2.41
Approach 2	−1.86	−7.05	2.69	−8.71	3.78
African/elsewhere					
Unweighted	0.58	7.97	13.93	19.52	23.08
Approach 1	−1.38	0.02	−0.27	−1.16	2.79
Approach 2	−0.30	−1.84	−3.16	−1.12	−0.89
Asian					
Unweighted	10.40	32.28	39.48	36.65	14.91
Approach 1	0.32	1.93	−3.54	1.42	1.14
Approach 2	8.81	4.64	3.68	3.69	3.64
Other ethnicities					
Unweighted	4.37	−5.08	−7.84	−2.35	6.90
Approach 1	3.31	−0.32	−1.80	2.43	−3.16
Approach 2	3.92	−2.52	−5.81	−0.65	−0.13

* standardized version of the covariate; BILAG, British Isles Lupus Assessment Group disease activity index; USA, United States of America.

REFERENCES

CRUMP, R. K., HOTZ, V. J., IMBENS, G. W. & MITNIK, O. A. (2009). Dealing with limited overlap in estimation of average treatment effects. *Biometrika* **96**, 187–99.

IMAI, K., KING, G. & STUART, E. (2008). Misunderstandings between experimentalists and observationalists about causal inference. *J. R. Statist. Soc. A* **171**, 481–02.

IMAI, K. & RATKOVIC, M. (2015). Robust estimation of inverse probability weights for marginal structural models. *J. Am. Statist. Assoc.* **110**, 1013–23.

IMAI, K. & VAN DYK, D. A. (2004). Causal inference with general treatment regimes: generalizing the propensity score. *J. Am. Statist. Assoc.* **99**, 854–66.

MOSCA, M., TANI, C., CARLI, L. & BOMBARDIERI, S. (2011). Glucocorticoids in systemic lupus erythematosus. *Clin. Exp. Rheumatol.* **29**, 126–29.

PETERSON, M. L., PORTER, K. E., GRUBER, S. G., WANG, Y. & VAN DER LAAN, M. J. (2010). Diagnosing and responding to violations in the positivity assumption. *Statist. Meth. Med. Res.* **21**, 31–54.

SMITH, V. A., PREISSER, J. S., NEELON, B. & MACIEJEWSKI, M. L. (2014). A marginalized two-part model for semicontinuous data. *Statist. Med.* **33**, 4891–30.
